# Dissolution Mechanism of YbOF in (LiF-CaF_2_)_eut._ Molten Salt

**DOI:** 10.3390/molecules30020425

**Published:** 2025-01-20

**Authors:** Linsheng Luo, Kailei Sun, Xu Wang

**Affiliations:** School of Metallurgical Engineering, Jiangxi University of Science and Technology, Ganzhou 341000, China; luo735373347@163.com (L.L.); skl0323@163.com (K.S.)

**Keywords:** molten salt systems, fluorine oxides, solubility, ab initio molecular dynamics, high-temperature Raman spectroscopy

## Abstract

The dissolution mechanism of YbOF in a fluoride-containing (LiF-CaF_2_)_eut._ molten salt is the basis for analyzing the structure of the resulting medium and optimizing the electrolytic preparation of rare-earth Yb alloys. In this study, isothermal saturation was used to analyze solubility changes of YbOF in the (LiF-CaF_2_)_eut_. system. Quantum chemical and molecular dynamics ab initio methods were used to study the basic properties of the components of the (LiF-CaF_2_)_eut._-YbOF system and the microscopic structural changes during the dissolution process. In addition, structural changes in the YbOF-saturated (LiF-CaF_2_)_eut._ system were analyzed by combining cryogenic-temperature Raman spectroscopy with experimental methods. The results show the solubility of YbOF increased linearly in the temperature range of 1073–1323 K. As the melting temperature exceeded 1073 K, LiF and CaF_2_ gradually dissociated into Li^+^, Ca^2+^, and F^−^. In the initial stages of YbOF dissolution (1073–1173 K), the Yb–F bond was less stable than the Yb–O bond; YbOF dissociated into YbO^+^ and F^−^ in this temperature range. When the temperature was increased above 1173 K, YbO^+^ further dissociated into Yb^3+^ and O^2−^. Overall, the dissolution of YbOF did not affect the main structure of the (LiF-CaF_2_)_eut._ system.

## 1. Introduction

Ytterbium is a variable-valent rare-earth element with broad applications in aluminum and magnesium alloys [1,2]. Because of its relatively high vapor pressure, Yb metal is generally produced using a vacuum reduction method, which is both costly and cumbersome [3,4]. Therefore, exploring low-cost processes for continuously preparing Yb alloys is theoretically and practically significant. Molten salt electrolysis, a common method used for the production of rare-earth metals and alloys, allows for the continuous, low-cost production of rare-earth metal products with uniform and controlled composition [5,6]. Some researchers have explored the feasibility of preparing rare-earth Yb alloys using active metal self-consuming cathode electrolysis in the MF-YbF_3_-Yb_2_O_3_ (M = Li, Ca) electrolyte system [7,8]. Exploring the dissolution of Yb_2_O_3_ in the LiF-YbF_3_ system to further improve the electrolysis efficiency and analyze the operative mechanism during electrolysis. Thermodynamic analyses indicate that Yb_2_O_3_ reacts preferentially in the LiF-YbF_3_ system to form YbOF (i.e., YbF_3_ + Yb_2_O_3_→3YbOF). Therefore, the study of the solvation mechanism of YbOF in fluoride molten salt systems is the basis for resolving the reactions involving Yb^3+^ that occur at the electrode. The main carrier for the preparation of rare earth metals and alloys by molten salt electrolysis is the fluoride-rare earth oxide system. The solubility of rare earth oxides in the molten fluoride is the most important property. However, solubility data at high temperatures are limited. Rare earth oxides in fluoride high-temperature molten salts may react to form rare earth fluorides, oxyfluorides, or other complex compounds, which involves interactions between different compounds, and accurately describing and predicting the dissolution mechanism is the main difficulty. Studying the dissolution mechanism of rare earth oxides in fluoride molten salts faces multiple challenges, such as high-temperature environments, complex chemical reactions, and experimental techniques.

In this study, we examined the effect of YbOF dissolution on the (LiF-CaF_2_)_eut._ architecture by combining freezing-point reduction and high-temperature Raman spectroscopy. Moreover, quantum chemistry and ab initio molecular dynamics calculations were used to study the components and structural properties of (LiF-CaF_2_)_eut._-YbOF. The theoretical calculations and experimental data were combined to analyze the dissolution mechanism of YbOF in the (LiF-CaF_2_)_eut._ molten salt system, as well as the structural changes and ionic composition of the system. These results provide a theoretical basis for further investigations on the cathodic reduction and alloying of Yb^3+^ ions. In this work, the dissolution mechanism of rare earth oxides is analyzed by a combination of molecular dynamics ab initio calculations and spectroscopic experimental studies, which can provide a better understanding of the dynamics of solubility and its influencing factors, and provide a basis for the optimal control of the electrolysis process parameters in the electrolytic production process of rare earth metals.

## 2. Experimental and Computational Methods

### 2.1. Solubility Measurements and High-Temperature Raman Spectroscopy Experiments

**Figure 1:**
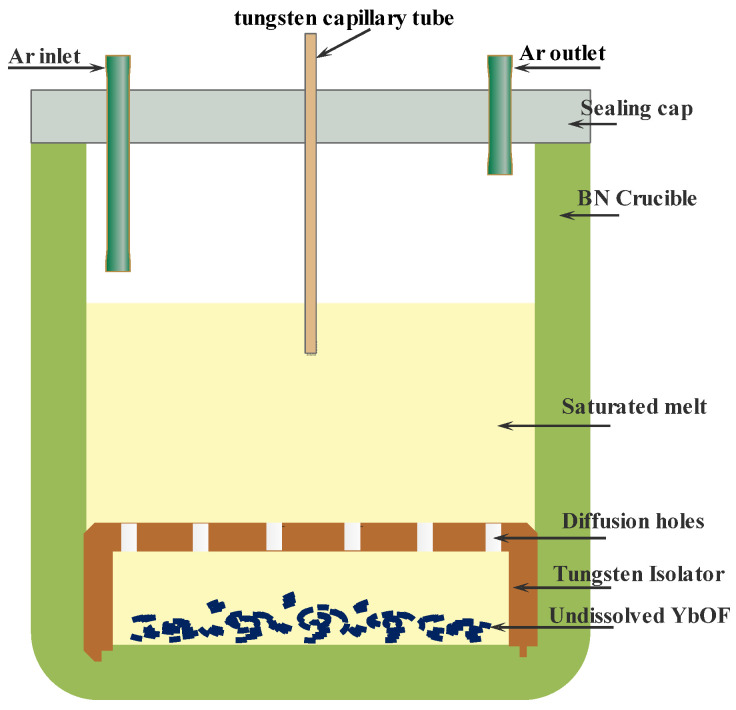
Schematic diagram of the solubility experiment device.

The solubility of YbOF in the (LiF-CaF_2_)_eut._ molten salt system was determined using the isothermal saturation method [9]. The raw materials were homogeneously mixed with a molar ratio of LiF:CaF_2_ = 79:21. YbOF (5 wt%) was then added, which was heated and melted under argon to the left for 120 min to form the upper saturated layer of the (LiF-CaF_2_)_eut._-YbOF molten salt. Subsequently, the supernatant of the molten salt was extracted every 20 min using a tungsten capillary tube, then cooled and analyzed by ICP–OES to determine the Yb content, which was used to calculate the solubility of YbOF. An apparatus was designed to measure the solubility of YbOF in the (LiF-CaF_2_)_eut._ molten salt system, as shown in Figure 1. A boron nitride crucible was used to carry the molten salt, and a pure tungsten barrier was placed at the bottom, with diffusion holes (diameter = 2 mm) at the top to ensure that the YbOF could diffuse smoothly from the tungsten barrier to the upper molten salt layer.

A Raman spectrometer equipped with a high-temperature stage was used to measure the Raman spectra of the (LiF-CaF_2_)_eut._ and (LiF-CaF_2_)_eut._-YbOF systems at varying temperatures (973, 1073, and 1173 K).

### 2.2. Freezing Point Depression Experiments for the (LiF-CaF_2_)_eut._-YbOF System

The decrease in initial crystallization temperature (ΔT) of the (LiF-CaF_2_)_eut._-YbOF (0.09–0.45 mol%) mixed molten salt system was determined by differential thermal analysis (DSC/TG, STA 449 F5) under an argon atmosphere. The solid-state samples were heated to the molten state at a rate of 20 K min^−1^, held for 30 min, and then cooled to 313 K at a rate of 5 K min^−1^; DSC curves of the samples were then obtained. The melting point of the molten salt system was determined using the extrapolated tangent method. Based on the principles of this method of freezing point depression [10,11], Equation (1) was used to calculate the number of nascent plasmas in the system and to analyze the mode of dissociation of YbOF in (LiF-CaF_2_)_eut._ molten salt medium:

(1) $$\Delta T=\frac{N_{1}\Delta N_{x}RT_{f}^{2}}{\Delta H_{f}},$$

where ΔT is the decrease in the incipient crystallization temperature of the (LiF-CaF_2_)_eut._ system (K); $\Delta N_{x}$ is the molar fraction of the added solute YbOF; R is the gas constant (8.314 J·mol^−1^·K^−1^); *T*_f_ is the melting temperature of the solvent (LiF-CaF_2_)_eut._ (K); $\Delta H_{f}$ is the enthalpy of melting of the solvent (LiF-CaF_2_)_eut._ (J·mol^−1^); and *N*_1_ is the number of newborn plasmas in the system.

### 2.3. Quantum Chemistry/Molecular Dynamics Computational Methods

The basic quantum chemical parameters for LiF, CaF_2_, and YbOF were calculated using the B3LYP [12] method by choosing a mixed basis group in which Li, Ca, F, and O atoms were used in the Pople basis group in 6-311G* [13,14], and Yb atoms were used in the SDD pseudopotential basis group. The 79 LiF, 21 CaF_2_, and 2 YbOF optimized molecules were randomly populated into a cubic box with a side length of 145.0 nm as a preprocessing model using Packmol (20.14.3) software [15,16]. The NPT_I system was used, and periodic boundary conditions were set. The temperature was controlled at 1123 K using a Nose-Hoover heat bath, and the pressure was set to 1.013 × 10^5^ Pa. Ab initio molecular dynamics (AIMD) calculations were performed based on the CP2K package [17,18], with density generalization based on the mixed Gaussian planar (GPW) basis set of the Quickstep module [19], and the orbitals described by an atom-centered Gaussian-type basis set. The orbital transformation (OT) method was used for self-concordant field (SCF) convergence with accuracy set to 10^−6^ Hartree. All atoms were described using the DZVP-MOLOPT-SR-GTH basis set [20]. All extra-nuclear electrons of the Li, Ca, F, and O atoms were assigned as valence electrons. The 3d, 4f, and 6s orbital electrons of the Yb atoms were assigned as valence electrons, and the remaining orbital electrons were described using PBE pseudopotentials. A Grimme-D3 dispersion correction was performed [21]. Geometric optimization was performed using the L-BFGS minimization algorithm with the convergence criteria listed in Table 1, a step size of 1 fs (TIMESTEP), 10,000 steps (STEP), and an operation time of 10 ps. The maximum value of the grid precision cutoff was set to 600 Ry, and REL_cutoff was set to 60 Ry [22]. Post-processing analyses were performed using the VMD (1.9.3) and VASTA (3.4.7) software.

## 3. Results and Discussion

### 3.1. Variable Law of Solubility of YbOF

**Figure 2:**
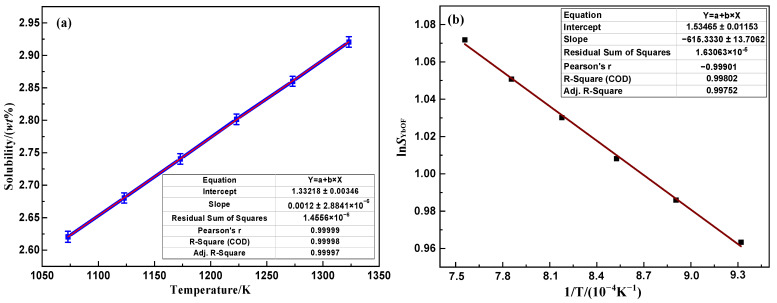
(**a**) Variation in YbOF solubility with temperature in the (LiF-CaF_2_)_eut._ molten salt system and (**b**) the relationship between ln*S*_YbOF_ and 1/T.

Figure 2a shows the variation rule of solubility of the YbOF in (LiF-CaF_2_)_eut._ molten salt system. The solubility data were analyzed by linear fitting to show that the solubility of YbOF increased linearly with increasing temperature in the range of 2.60–2.90 wt% in the temperature range of 1073–1323 K, in accordance with Equation (2). Based on the fact that the solubility of YbOF in the (LiF-CaF_2_)_eut._ system exhibits a stable linear increasing relationship, it can be hypothesized that the dissolution process of YbOF in the (LiF-CaF_2_)_eut._ medium does not undergo a drastic chemical interaction. Based on the solubility data, the relationship between ln*S*_YbOF_ and 1/T can be obtained as Equation (3) and shown in Figure 2b and the free energy of dissolution of YbOF, $\Delta G_{YbOF}^{\theta }$, and the activity coefficient, $\gamma_{YbOF}$, can be further estimated to be about 7413.16 J·mol^−1^ and 5.12, respectively.

(2) $$S_{YbOF}=1.33+1.20\times 10^{-3}T$$

(3) $${lnS}_{YbOF}=\;-\frac{\Delta G_{YbOF}^{\theta }}{RT}+ln\gamma_{YbOF}$$

where *S*_YbOF_ is the solubility of YbOF in (LiF-CaF_2_)_eut._ system, (wt%); *T* is the temperature (K); $\Delta G_{YbOF}^{\theta }$ is the free energy of dissolution of YbOF (J·mol^−1^); $\gamma_{YbOF}$ is the activity coefficient of YbOF.

### 3.2. Analysis of Dissolution Reactions

**Figure 3:**
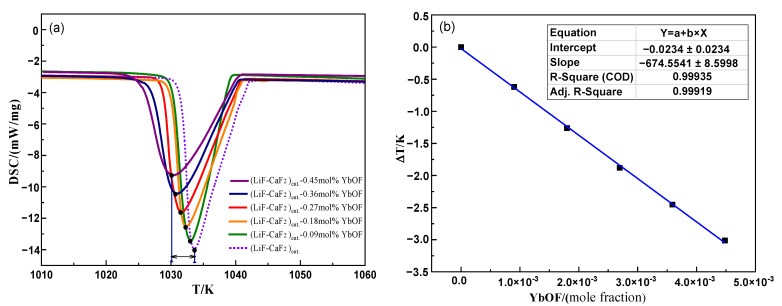
(**a**) Influence of YbOF concentration on the DSC curve of the (LiF-CaF_2_)_eut._ system and (**b**) variation in melting point depression.

Based on the solubility data for YbOF in Figure 1, the DSC curve of the unsaturated (LiF-CaF_2_)_eut._-YbOF molten salt system with YbOF molar percentages ranging from 0.09% to 0.45% (corresponding to YbOF mass fractions of 0.5–2.5%) is shown in Figure 3a. It can be seen that the melting point of the (LiF-CaF_2_)_eut._-YbOF molten salt system decreased with increasing YbOF content. Furthermore, the freezing point depression (ΔT) of the system decreased linearly with the molar fraction of added YbOF ($\Delta N_{x}$), and the slope of the fitted straight line [$\Delta T/\Delta N_{x}$] was approximately –674.6, as shown in Figure 3b. Thermal analysis indicated that the unsaturated (LiF-CaF_2_)_eut._-YbOF system could be approximated as ideally mixed. The enthalpy of melting (Δ*H_f_*) of the (LiF-CaF_2_)_eut._ system was found to be 22 022 J·mol^−1^ by product fitting the heat absorption peak (Figure 2a). According to the freezing point depression Equation (1), the number of newly generated plasmas *N*_1_ ≈ 1.675 (between 1 and 2) can be calculated from the dissolution of YbOF, and it can be surmised that there are three possible forms of dissolution after the introduction of a YbOF into the (LiF-CaF_2_)_eut._ solvent, as shown in Equations (4)–(6). Dissolved form (4) is the product of Yb–F bond cleavage in YbOF to afford YbO^+^ plasma. Solvated form (5) is the combination of YbOF and fluoride ions to form an ionic luster. When *n* = 0 in Equation (4), YbOF is dissolved in its physical form in the (LiF-CaF_2_)_eut._ system, whereas when *n* > 0 in Equation (5), the newly generated ionic cluster contains only one Yb atom with an empirical formula of [YbOF_n_]^1−n^. Solvated form (6) comprises Yb^3+^, O^2−^, and F^−^ ions from Yb–F and Yb–O bond cleavage in YbOF.


YbOF → YbO^+^ + F^−^ (4)

YbOF + nF^−^ → [YbOF*_n_*]^1−*n*^ (5)

YbOF → Yb^3+^ + O^2−^ + F^−^ (6)

### 3.3. Dissolution Component Quantum Chemistry/Molecular Dynamics Analysis

**Figure 4:**
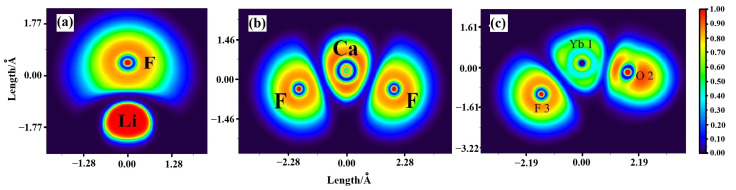
Projection maps of (**a**) LiF, (**b**) CaF_2_, and (**c**) YbOF-localized orbitals.

The electron localization function projection maps of LiF, CaF_2_, and YbOF are shown in Figure 4. By analyzing the color-coded values (ranging from 0 to 1; ELF = 1 indicates complete electron localization, ELF = 0 indicates complete electron delocalization, and ELF = 0.5 indicates free electron gas), the distribution and states of electrons in LiF, CaF_2_, and YbOF can be elucidated. The inner-shell electrons of Li^+^ and F^−^ in LiF are completely localized, whereas the outer-shell electrons near the Li–F bond critical point (BCP) are delocalized, indicating ionic bonding between Li and F. In CaF_2_, the inner-shell electrons of the F atoms show a weaker tendency toward localization compared to that of the Ca atoms, and the electrons near the Ca–F BCP are completely delocalized, indicative of ionic bonding between Ca and F. Finally, the inner-shell electrons of the F and O atoms in YbOF are completely localized with an ELF value near 0 at the BCP between the Yb–F and Yb–O bonds, indicating complete electron delocalization and ionic bonding between Yb and F, as well as Yb and O.

The ionic bond strength and molecular polarity in LiF, CaF_2_, and YbOF are the main parameters for analyzing their high-temperature dissolution and ionization processes. Meanwhile, the distribution of the molecular surface electrostatic potential (EPS) can be used to analyze the electrostatic interactions between individual molecules in the melt [23,24], thus providing a deep analysis of the dissolution process in the high-temperature condensed state of the (LiF-CaF_2_)_eut._-YbOF molten salt system.

**Figure 5:**
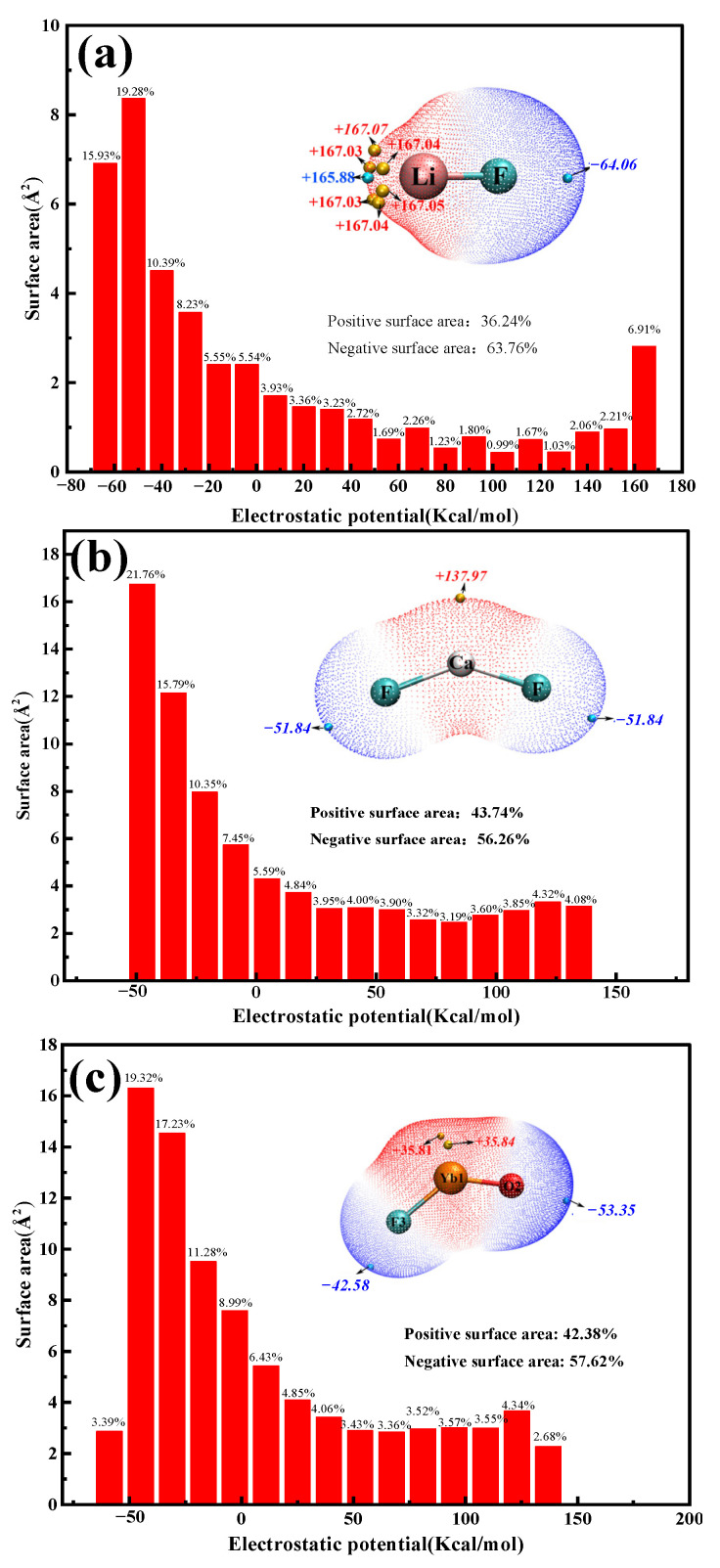
Statistical plots of the van der Waals surface electrostatic potential and area of potential intervals for: (**a**) LiF, (**b**) YbF_3_, and (**c**) YbOF.

The Relaxed Force Constant [25] is the second-order derivative of the potential energy of the system with respect to the bond length when the bond is in the equilibrium position, which reflects the curvature of the potential energy surface in the direction of the bond, and it can be used to make a side-by-side comparison between different types of bonds within different systems. Moreover, it is an objective way to measure the bond strength. In this study, the flexible force constant and bond length parameters were used to analyze the bond strengths in individual molecules in the system. Additionally, the Molecular Polarity Index (MPI) [26], based on the van der Waals (VDW) surface electrostatic potential, was used for quantitative polarity analysis of the molecules in the molten salt system, as shown in Table 2 and Table 3. The data in Table 2 show that the order of the flexible force constants between the ionic bonds in the system ranges such that [Ca–F] < [Yb–F] < [Li–F] < [O–Yb], in which the Ca–F, Yb–F, and Li–F bond strengths are similar to one another and are susceptible to cleavage at high temperatures, whereas the Yb–O bond is more stable and is capable of forming YbO^+^ cations at high temperature. However, when comparing the bond length data in Table 2, the order of bond strength should be [Li–F] > [Yb–O] > [Ca–F] > [Yb–F], which is inconsistent with the results from the flexural force constants, suggesting that comparison of bond lengths in ionic solids does not realistically reflect bond strengths. The MPI data in Table 3 show that the polarity of LiF, CaF_2_, and YbOF is in the order of LiF > CaF_2_ > YbOF. Additionally, the polar surface area constitutes over 85% of the total surface area for these compounds. This high polarity suggests that strong ionic interactions occur during the dissolution process. A molecular surface analysis method [27] was used to locate the extreme values and minima of the electrostatic potential on the van der Waals (VMD) surface. The distribution of different electrostatic potential intervals on the van der Waals surface was quantitatively calculated, and the results are shown in Figure 4 (*ρ* = 0.001 Arb. Units isosurfaces). The ESP values on the surface of the VMD in LiF, CaF_2_, and YbOF (kcal mol^−1^) were found in the range of [–64.06, +167.07], [–51.84, +137.97], and [–53.55, +35.84], respectively. The ESP values span more than 80 kcal mol^−1^, indicating that the free states of LiF, CaF_2_, and YbOF exhibit strong electrostatic interactions between cationic and anionic charges. The ESP minima are located on the surfaces of the F and O atoms, which are more accessible to other polar molecules on the electrophilic surface. LiF has six extremely large points of simplicity, and all of them are located on the surface of Li atoms, which renders the approach toward nucleophilic surfaces of other polar molecules more facile. The maxima of the ESPs for CaF_2_ and YbOF are located on one side of the surface in proximity to Ca and Yb atoms, respectively, suggesting that Ca and Yb are more likely to interact with the electrophilic surfaces (maxima of ESP) of other polar molecules. The surface areas of LiF, CaF_2_, and YbOF with different electrostatic potential intervals, as shown in Figure 5, were determined to be 36.24, 43.74, and 42.38% of the positive electrostatic potential regions of LiF, CaF_2_, and YbOF, respectively. Moreover, the corresponding negative electrostatic potential regions of LiF, CaF_2_, and YbOF accounted for 63.76%, 56.26%, and 57.62%. Therefore, the surface electrophilic interaction region is larger than the nucleophilic interaction region. The data in Table 2 show that the absolute value of the average ESP positive region, |${\bar{V}}_{s}^{+}$|, on the VMD surfaces of LiF, CaF_2_, and YbOF is higher than that of the corresponding negative region, and the variance in the ESP positive value, $\sigma_{+}^{2}$, on the surfaces of the corresponding ionic compounds is much higher than the variance in the ESP negative value, $\sigma_{-}^{2}$, which indicates that the ESP positive regions of LiF, CaF_2_, and YbOF molecules are more prone to interactions than the nucleophilic region.

Structural modifications to the system can be analyzed from the change in the atomic spacing in the high-temperature (LiF-CaF_2_)_eut._-YbOF molten salt system. To ensure that the temperature and potential energy of the saturated (LiF-CaF_2_)_eut._-YbOF system reached equilibrium, we based all analyses in this study on the calculated molecular dynamics trajectory data between 2000–9000 fs.

**Figure 6:**
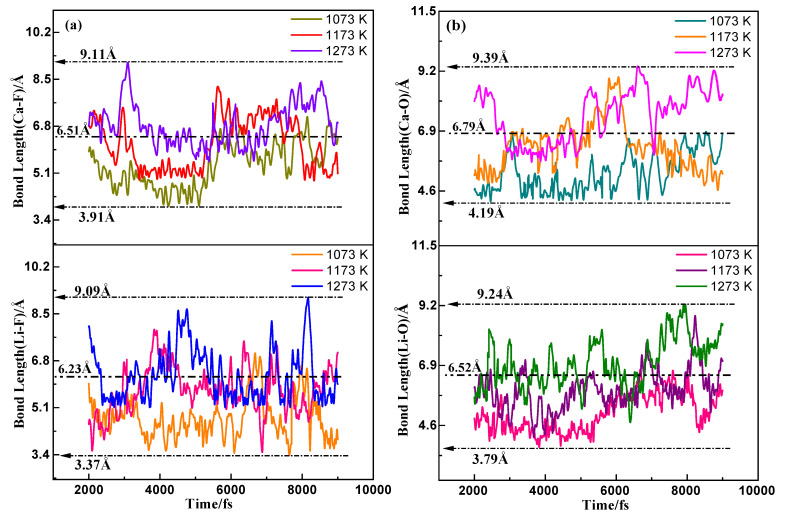
Curves of atomic spacings for (**a**) [Li–F], [Ca–F] and (**b**) [Li–O], [Ca–O] in the (LiF-CaF_2_)_eut._-YbOF system at varying temperatures.

**Figure 7:**
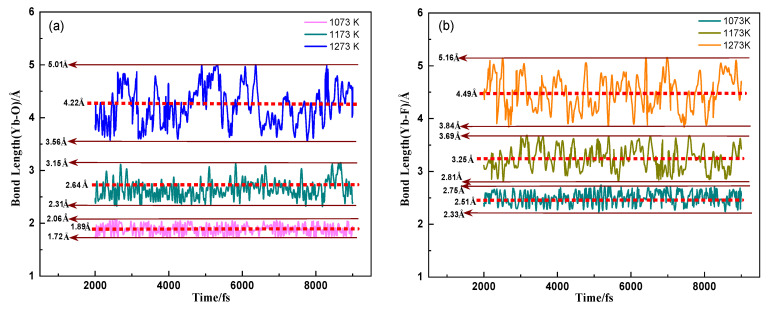
Curves of (**a**) [Yb–O] and (**b**) [Yb–F] atomic spacings in dissolved YbOF at varying temperatures.

The maximum, minimum, and average values of the interatomic spacing of Li, Ca, and F atoms in the (LiF-CaF_2_)_eut._ system and O atoms in the dissolved YbOF at the three melting temperatures (1073, 1173, and 1273 K) are shown in Table 4 and were calculated by AIMD. Figure 6a,b display the variation curves of interatomic distances corresponding to [Li–F], [C–F], [Li–O], and [Ca–O] atomic pairs from 2000 fs to 9000 fs, as listed in Table 4. The analysis of the data in Table 3 and Figure 5 shows that the maximum, minimum, and average values of the [Li–F] atomic spacing in the (Li-CaF_2_)_eut._ medium increase gradually with increasing temperature. Additionally, the fluctuating range of the [Li–F] atomic pair spacing also increases gradually, with the average value (6.23 Å) and the fluctuating range (3.37–9.09 Å) between 1073 and 1273 K. These measurements are significantly larger than the bond length of 1.81 Å for LiF, as calculated by quantum chemistry (1.81 Å), despite LiF being fully ionized. Meanwhile, the [Ca–F], [Li–O], [Ca–O], and [Li–F] atom pair spacings show the same variation with temperature. The [Li–O] and [Ca–O] pair spacing and fluctuation range are slightly higher than the corresponding values for [Li–F] and [Ca–F], indicating that the O atoms in YbOF are also fully ionized and interacting with Li and Ca atoms in the medium. Figure 7a,b shows the variation curves of the [Yb–O] and [Yb–F] atomic spacings in the dissolved YbOF in (LiF-CaF_2_)_eut._ at temperatures of 1073, 1173, and 1273 K, respectively. The variation of [Yb–O] atomic spacing at 1073 K ranges from 1.72–2.06 Å with an average value of 1.89 Å, which is close to the quantitatively calculated value of 1.834 Å. The fluctuation of [Yb–F] atomic spacing ranges from 2.33–2.75 Å with an average value of 2.51 Å, which is much higher than that of the quantitatively calculated value of 2.023 Å. This suggests that the Yb–O bond of YbOF solubilized in the (LiF-CaF_2_)_eut._ medium remains stable, whereas the Yb–F bond dissociates. Combined with the results of the freezing-point depression analyses, it was assumed that dissolved YbOF takes on a solvated form (1) listed above. Further comparison of the changes in the interatomic spacing between [Yb–O] and [Yb–F] at 1173 and 1273 K shows that the range of fluctuation in the interatomic spacing continues to increase with increasing temperature, with the average value being much higher than that quantitatively calculated, indicating that the Yb–O and Yb–F bonds are in a dissociated state and that YbOF is solubilized in solvated form (3) listed above when dissolved in the (LiF-CaF_2_)_eut._ medium.

**Figure 8:**
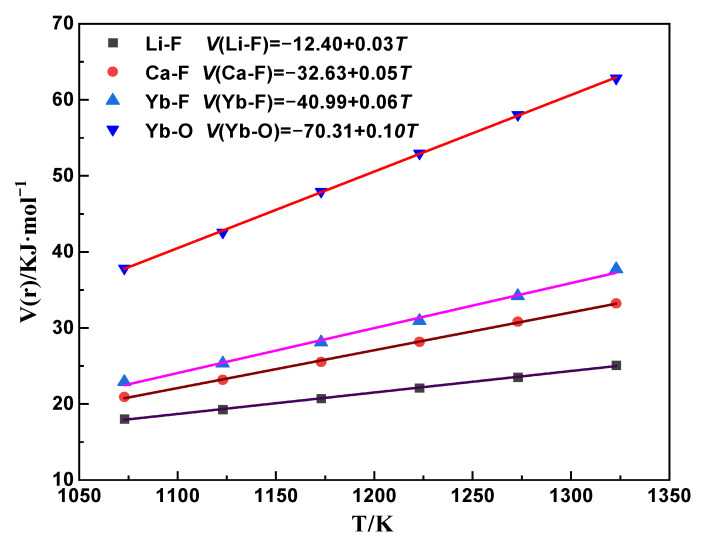
Interatomic migration energy barriers for [Li–F], [Ca–F], [Yb–F], and [Yb–O] in the (Li-F-CaF_2_)_eut._-YbOF system.

The migration energy barriers of the [Li–F], [Ca–F], [Yb–F], and [Yb–O] interatomic interactions in the (LiF-CaF_2_)_eut._-YbOF system are shown in Figure 8 and lie in the range of 1073–1323 K. It shows that the migration energy barriers at the same temperature are in the order of [Yb–O] > [Yb–F] > [Ca–F] > [Li–F], and the migration energy barrier of the Yb–O bond is much higher than the dissociation energy barriers of other the atomic pairs. Moreover, stable YbO^+^ ions are formed in the system, while the Yb–F, Ca–F, and Li–F bonds dissociate into ions at the corresponding temperatures.

### 3.4. High-Temperature Raman Spectroscopic Analysis of the YbOF Dissolution Process

**Figure 9:**
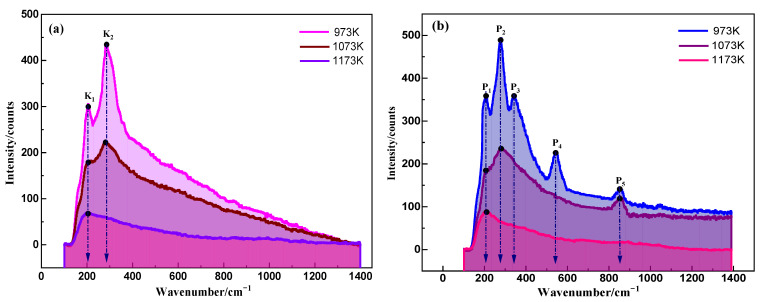
Raman spectra of (**a**) the (LiF-CaF_2_)_eut._ system and (**b**) the (LiF-CaF_2_)_eut._-YbOF system in a saturated state at varying temperatures.

Raman spectra of (LiF-CaF_2_)_eut._ and the YbOF saturated systems at varying temperatures (973, 1073, and 1173 K) are shown in Figure 9a and Figure 9b, respectively.

Figure 9a shows that the (LiF-CaF_2_)_eut._ system has two Raman spectral peaks, K_1_ (≈205 cm^−1^) and K2 (≈280 cm^−1^) at 973 K. According to the phase diagram of the LiF-CaF_2_ system, the system is in the solid-state at 973 K, and the component atoms are in a thermally vibrating state at the equilibrium position. LiF is a linear molecule that belongs to the $C_{\infty v}$ point group and exhibits only one simple positive stretching vibration A_1_ (3N–5, where N is 2) and one Raman-active vibration as both the polarization rate and dipole moment change during the vibration. CaF_2_ is a nonlinear molecule belonging to the C_2v_ point group, and there are three simple positive vibrations (3N–6, where N is 3): symmetric telescopic vibrations (A_2_), asymmetric telescopic vibrations (B), and torsional vibrations (S) of two F atoms centered on the Ca atoms, all of which are non-simple merging vibrations that are Raman-active.

Since the torsion vibration (S) of CaF_2_ has the highest energy, followed by the asymmetric stretching vibration (B), the corresponding Raman spectral peaks are attributed to K_1_→B and K_2_→S, while the simple ortho-stretching vibration A_1_ of the Li–F bond and the symmetric stretching A_2_ of the F–Ca–F moiety have lower energies, which are superimposed in the background of the Raman spectral pattern. When the temperature reached 1073 K, the (LiF-CaF_2_)_eut._ system became liquid, and the main composition was F^−^, Ca^2+^, and Li^+^, leading to the Raman activity for K_1_ and K_2_ at 973 K being considerably attenuated and the peaks being rounded and smoothed out, indicative of transient polarization regions in the melt in the short-range microregions of F^−^, Ca^2+^, and Li^+^. When the temperature was increased further to 1173 K, the ionic kinetic energy of the (LiF-CaF_2_)_eut._ system increased, resulting in a fully ionized state. Concurrently, the “smooth” peaks in the Raman spectra decayed to less than 100 cm^−1^, indicating weak Raman activity. This observation suggests that the local energy fluctuations within the melt are responsible for triggering the observed weak Raman activity. Figure 9b shows the Raman spectrum of the (LiF-CaF_2_)_eut._-YbOF system at 973 K, which contains five peaks: P_1_ (≈205 cm^−1^), P_2_ (≈280 cm^−1^), P_3_ (≈348 cm^−1^), P_4_ (≈547 cm^−1^), and P_5_ (≈860 cm^−1^), in which peaks P_1_ and P_2_ are nearly the same wavenumbers as the peaks K_1_ and K_2_ in Figure 9a, which belong to the torsion vibration (S) and asymmetric stretching vibration (B) of CaF_2_. YbOF is nonlinear (*C*_1_ point group), and there are three simple orthogonal vibrations (3N–6, where N is 3), which are the symmetric stretching (A_3_), asymmetric stretching (B_1_), and torsional vibration (S_1_) between F and O atoms centered on Yb; all of the vibrations are non-simplex vibrations and Raman-active. The torsion vibration (S_1_) of the YbOF is the highest in energy, corresponding to the Raman peak of P_3_. The asymmetric stretching vibration B_1_ is the next highest, corresponding to the Raman peak P_4_, and the lowest vibrational energy symmetric stretching A_3_ is attributed to P_5_. When the temperature reached 1073 K, the (LiF-CaF_2_)_eut._-YbOF system entered a liquefied ionic state, and the Raman activities of peaks P_1_ and P_2_ decayed dramatically at the corresponding temperature of 973 K. YbOF dissociated primarily into YbO^+^ and F^−^, while the corresponding Raman peaks P_3_ and P_4_ disappeared. Furthermore, the Raman peak P_5_ was mainly associated with the stretching vibration of the Yb–O bond. As the temperature was further increased to 1173 K, the (LiF-CaF_2_)_eut._-YbOF system was completely ionized, and YbOF mainly dissociated into Yb^3+^, O^2+^, and F^−^, corresponding to the disappearance of the P_3_, P_4,_ and P_5_ bands. The Raman spectra are essentially the same as that shown in Figure 9a, which also demonstrates weak Raman activity due to localized energy undulation of the melt.

A comparison of the number of vibrational peaks and vibrational intensities in the Raman spectra in Figure 9a,b shows that with increasing temperature, LiF and CaF_2_ in (LiF-CaF_2_)_eut._ dissociate into Li^+^, Ca^2+^, and F^−^, and the process of dissociation of YbOF is divided into two stages when the temperature is increased gradually during solubilization: YbOF → YbO^+^ + F^−^ → Yb^3+^ + O^2−^ + F^−^.

## 4. Conclusions

The solubility of YbOF in the (LiF-CaF_2_)_eut._ system is in the range of 2.60–2.90 wt% between 1073 and 1323 K and increases linearly with the temperature. There are three solubilized forms of YbOF in the (LiF-CaF_2_)_eut._ medium, which are: (i) YbOF → YbO^+^ + F^−^, (ii) YbOF + nF^−^ → [YbOF*_n_*]^1−*n*^, and (iii) YbOF → Yb^3+^ + O^2−^ + F^−^. In the range of 1073–1323 K, the migration energy barriers of the interatomic interactions in the (LiF-CaF_2_)_eut._-YbOF system are ordered as follows: [Yb–O] > [Yb–F] > [Ca–F] > [Li–F]. The migration energy barriers of the Yb–O bond are much higher than the dissociation energy barriers of the other pairs of atoms, and the YbO^+^ ion demonstrates superior stability, while the Yb–F, Ca–F, and Li–F bonds dissociate more easily into ionic states. As the melting temperature exceeded 1073 K, LiF and CaF_2_ gradually dissociated into Li^+^, Ca^2+^, and F^−^. Dissolution of YbOF in the (LiF-CaF_2_)_eut._ system is divided into two phases (i.e., YbOF → YbO^+^ + F^−^ → Yb^3+^ + O^2−^ + F^−^). In the early stages of dissolution (1073–1173 K), the Yb–F bond was less stable and, therefore, weaker than the Yb–O bond, and YbOF dissociated into YbO^+^ and F^−^. When the temperature exceeded 1173 K, YbO^+^ dissociated into Yb^3+^ and O^2−^, and the dissolution of YbOF did not affect the main structure of (LiF-CaF_2_)_eut._ system. The results of this study can provide a theoretical basis for optimizing the kinetic conditions for the dissolution of oxides of fluorine (YbOF) in fluoride systems.

## Figures and Tables

**Table 1:** Convergence criterion of geometric optimization (a.u).

Convergence Terms	MAX_DR	RMS_DR	MAX_FORCE	RMS_FORCE
Convergence Criteria Values	3 × 10^−3^	1.5 × 10^−3^	4.5 × 10^−4^	3 × 10^−4^

**Table 2:** Flexible force constants and bond lengths of the ionic bonds in LiF, CaF_2_, and YbOF.

Ionic Bonds	Li–F	Ca–F	Yb–F	Yb–O
Bond Lengths (Å)	1.810	1.991	2.023	1.834
RFC (mdyn·Å^−1^)	2.809	2.618	2.725	3.817

**Table 3:** Molecular Polarizability Parameters for LiF, CaF_2_, and YbOF.

Molecule	PSA	MPI (kcal·mol^−1 ^)	${\bar{V}}_{s}^{+}$	${\bar{V}}_{s}^{-}$	$\sigma_{+}^{2}$	$\sigma_{-}^{2}$
LiF	91.96%	57.85	83.46	–43.30	3462.30	306.98
CaF_2_	90.33%	48.11	67.29	–33.19	1818.20	204.69
YbOF	87.97%	44.73	62.74	–31.48	1867.48	215.74

**Table 4:** Li, Ca, F, and O atomic spacings in the (LiF-CaF_2_)_eut._-YbOF system at varying temperatures.

	Li–F	Ca–F	Li–O	Ca–O
Temperature (K)	1073 1173 1273	1073 1173 1273	1073 1173 1273	1073 1173 1273
Max (Å)	7.07 7.93 9.09	7.14 8.24 9.11	6.75 8.81 9.24	6.88 8.98 9.39
Min (Å)	3.37 3.48 5.10	3.91 4.82 5.51	3.79 4.14 4.71	4.19 4.62 5.71
Mean (Å)	4.73 5.72 6.27	5.38 6.14 6.84	5.06 5.95 6.97	5.26 6.32 7.50

## Data Availability

Data are contained within the article.
